# Targeting Wnt/β-Catenin Pathway for Developing Therapies for Hair Loss

**DOI:** 10.3390/ijms21144915

**Published:** 2020-07-12

**Authors:** Bu Young Choi

**Affiliations:** Department of Pharmaceutical Science & Engineering, Seowon University, Cheongju, Chungbuk 28674, Korea; bychoi@seowon.ac.kr; Tel.: +82-43-299-8411; Fax: +82-43-299-8470

**Keywords:** hair loss, Wnt/β-catenin signaling

## Abstract

Persistent hair loss is a major cause of psychological distress and compromised quality of life in millions of people worldwide. Remarkable progress has been made in understanding the molecular basis of hair loss and identifying valid intracellular targets for designing effective therapies for hair loss treatment. Whereas a variety of growth factors and signaling pathways have been implicated in hair cycling process, the activation of Wnt/β-catenin signaling plays a central role in hair follicle regeneration. Several plant-derived chemicals have been reported to promote hair growth by activating Wnt/β-catenin signaling in various in vitro and in vivo studies. This mini-review sheds light on the role of Wnt/β-catenin in promoting hair growth and the current progress in designing hair loss therapies by targeting this signaling pathway.

## 1. Introduction

Hair loss is characterized by either the loss of hair density or hair thinning, or both, and it may result from both hormonal and non-hormonal causes. The loss of hair caused by the excess responsiveness of testosterone and its active metabolite dihydrotestosterone is known as androgenic alopecia, which has been extensively investigated for the understanding of its pathophysiology and designing therapies for hair growth promotion. Non-hormonal causes of hair loss include aging, scalp inflammatory disorders, auto-immune disorder, and mechanical stress, which are known as senescent alopecia, cicatricial alopecia, alopecia areata, and traction alopecia, respectively. Focal hair loss, resulting secondarily from an underlying medical disorder, may either be non-scarring patchy hair loss, such as tinea captitis, alopecia areata, or may be a rare scarring type cicatricial alopecia, for example hair loss resulting from lupus erythematosus [1]. On the other hand, the diffuse type hair loss is characterized by hair thinning commonly presented in telogen effluvium, anagen effluvium, and androgenic alopecia [2]. Among the non-scarring or non-cicatricial alopecia, alopecia areata is largely a patchy-type hair loss that is thought to result from autoimmunity and is characterized by the presence of vellus hair with broken shaft. A rarer diffuse type alopecia areata has also been reported. The alopecia areata confined to the entire scalp is called alopecia totalis, whereas a loss of whole body hair is known as alopecia universalis. The androgenic alopecia often has a family history and affects both male and female with distinct pattern of diffuse hair loss. The androgenic alopecia in men is represented as bitemporal thinning of the frontal and vertex scalp, whereas that in women is manifested by thinning of the vertex hair with loss in the frontal hairline. However, cases with preservation of frontal hairline and the appearance of a Christmas tree pattern have been reported [3].

Anagen effluvium is a diffuse non-scaring hair loss mostly associated with the use of chemotherapeutic agents, while telogen effluvium is linked with stress and involves loss of clumps of hair during hair brushing. Tinea capitis is also a patchy type hair loss that is associated with a dermatophyte infection and commonly occurs in children [4]. Irrespective of its etiology and type, the emotional stress caused by hair loss affects the quality of life of the afflicted people. For both male and female, scalp hair possesses an aesthetic value Progressive hair loss leading to terminal baldness is considered by an individual as a disgraceful condition and is a critical issue for social stigma, psychological distress, and often an indication of certain pathological conditions. Thus, the diagnosis and treatment of hair loss is crucial from the perspective of human health and wellness. 

Several lines of therapeutic modalities are currently available to promote hair re-growth or to prevent hair loss. These include the use of 2% or 5% topical minoxidil for androgenic alopecia and anagen effluvium; triamcinolone acetonide for alopecia areata; and antifungal agents (e.g., itraconazole, griseofulvin, or fluconazole) for tinea capitis. Finasteride, which blocks the conversion of testosterone to dihydrotestosterone, is also used to treat androgenic alopecia in patients unresponsive to minoxidil. However, these drugs have limited therapeutic efficacy and possess serious side effects [5,6,7,8]. For example, the use of minoxidil helps in hair regrowth but the effects are not long lasting and require prolonged use of the medication. Male patients using finasteride may experience adverse effects, such as loss of libido, erectile dysfunction, and gynecomastia [9]. Likewise, antifungal agents are known to affect the normal liver function. A randomized clinical trial demonstrated that the microinjection of adipose-derived mesenchymal stem cells (AD-MSCs) and stromal vascular fraction cells (SVFs) can stimulate the scalp epidermal stem cells and hair growth in androgenic alopecia patients. However, in many of these patients, frequent microinjection is required to sustain the hair growth [10]. Thus, novel therapies for hair loss treatment has long been sought. Interestingly, over the last several decades, there have been remarkable progress in understanding the molecular mechanisms of hair morphogenesis and the pathologic basis of hair loss. The inter- and intracellular signaling pathways including, but not limited to, sonic hedgehog, Wnt/β-catenin, bone morphogenetic protein (BMP), transforming growth factor (TGF)-β, and notch signaling, have been implicated in hair regeneration process. Efforts are made to target these signaling pathways to design novel therapies for alopecia. This review sheds light on the role of Wnt/β-catenin signaling in hair regeneration and the current research in developing therapy for hair loss by targeting this signaling pathway.

## 2. Hair Morphogenesis and Cycling

The hair follicle (HF) is an integral part of the skin that protects the internal organs and plays a role in body temperature regulation. HFs are specialized organelles that are constantly recycling. In general, a human scalp has about 120,000 HFs, which regulates the hair cycling and the constant maintenance of scalp hair. Structurally, HF extends from the epidermis into the deeper layer of the dermis where it takes a tubular shape making a bulb-like structure at the base. This follicular bulb encircles the dermal papillae (DP), which is composed of dermal papillary cells (DPC), connective tissues, and a capillary network. The DPCs constitute a special mesenchymal component that regulates periodic regeneration of HF [11]. A layer of hair matrix keratinocytes surrounds the lower DP. During the hair growth cycle, these keratinocytes undergo proliferation, thereby resulting in the formation of hair fiber. Moreover, hair follicle stem cells (HFSCs) are present in the bulge region of the HF [12,13,14]. These bulge region HFSCs can give rise to HF progenitor/stem cells, which further migrate down and regenerate HFs [15]. The depletion of these stem cells may lead to the development of scarring alopecia, while the maintenance of stem cells and loss of the progenitor cells are associated with baldness [16]. 

The hair shaft is originated from the follicular epithelial matrix. The three distinct stages of hair development are induction, organogenesis, and cytodifferentiation. The induction phase involves the thickening of epithelial cells to form a placode, followed by the organogenesis stage, when the epithelial cells first send signals to dermal cells to proliferate and form dermal condensate. This dermal condensate then directs epithelial cells to proliferate and migrate towards dermis. During cytodifferentiation stage, the dermal condensate forms the dermal papilla upon being encapsulated by the follicular epithelial cells. Several intra- and intercellular signaling molecules including various morphogens and growth factors play a key role in generating the HF [17].

**Figure 1:**
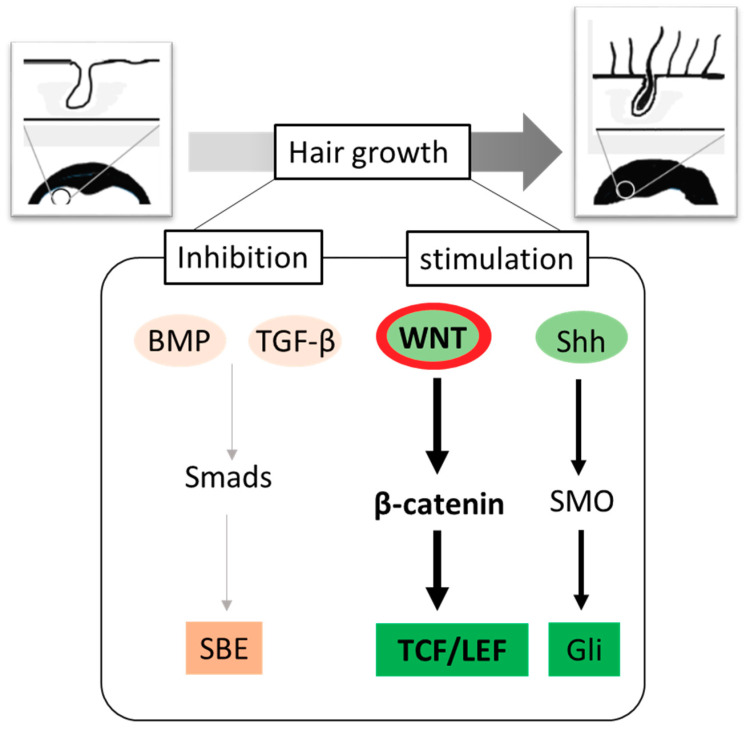
Molecular switches regulating hair growth. A variety of inter- and intracellular signaling molecules play critical roles in the formation of new hair. The negative regulators of hair growth include BMP and TGF-β signaling, whereas Wnt and Shh signaling activates anagen entry. The Wnt-mediated hair regrowth involves the stabilization of hypophosphorylated β-catenin, which interacts with TCF/LEF and translocates to nucleus to promote transactivation of growth promoting genes. Abbreviations: BMP, Bone morphogenetic proteins; TGF-β, Transforming growth factor-β; Smad, homologs of the Drosophila protein, mothers against decapentaplegic (Mad) and the *Caenorhabditis elegans* protein Sma; SBE, Smad-binding element; Wnt, wg derived from the Drosophila gene wingless (wg) and int derived from the proto-oncogene integration-1; TCF/LEF, T-cell factor/lymphoid enhancer factor; Shh, Sonic hedgehog; SMO-smoothened; Gli, glioma-associated oncogene family zinc finger 1.

The human hair cycle consists of three phases: the anagen, catagen, and telogen phases. The anagen phase that lasts between two and eight years is the active hair growth phase, whereas the catagen is the regression phase that ranges 2–3 weeks. The telogen is an about three-month resting phase that ends through the original hair falling out followed by the appearance of new hair [18,19]. A synchronized intercellular signaling between adjacent epithelial and mesenchymal cells leads to the formation and maintenance of the hair follicle. Until now, various endogenous factors, including both inhibitory and stimulatory signaling cascades, have been implicated in the hair cycling process (Figure 1). For example, BMP signaling is known to inhibit anagen induction [15], while Wnt10b promotes telogen to anagen re-entry of the HF [20]. Likewise, fibroblast growth factor (FGF) has been reported to induce anagen phase in telogenic C57BL/6 mice [21]. Although HF development occurs predominantly during the embryonic life, over- and/or under-activation of signaling pathways, such as sonic hedgehog (shh), notch, TGF-β, and Wnt/β-catenin, which regulate epithelial–mesenchymal interaction in HF, play roles in periodic regeneration of HF and, hence, hair cycling in adult [22]. Over the last several decades, extensive research into the understanding of highly intricate nature of these diverse signaling pathways has led to the unfolding of the pathophysiology of hair loss. Thus, attempts have been made to target one or more of these signal cascades to develop therapies for alopecia.

## 3. Role of Wnt/β-Catenin Signaling in Hair Growth

**Figure 2:**
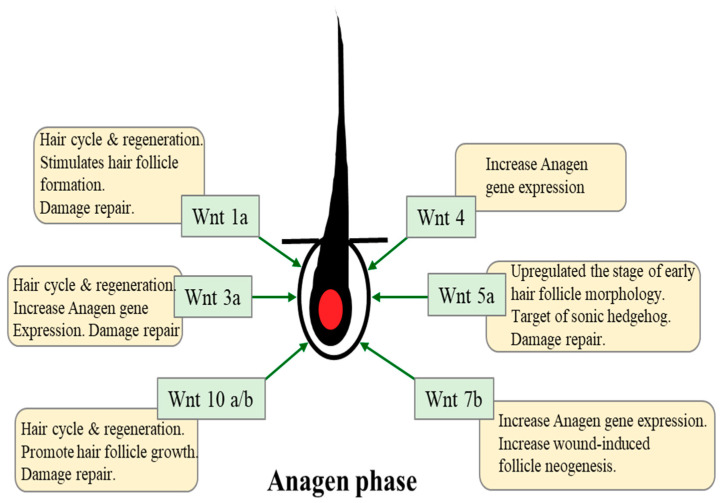
The role of various Wnts in hair growth cycle. Among the many signaling pathways implicated in HF development and growth, Wnt/β-catenin signaling in dermal papilla (DP) cells plays a critical role in mutual communication between DP and epithelial cells. The different types of Wnt play variable roles in HF morphogenesis and regeneration.

The hair development process requires a controlled cross talk between the mesenchymal and epithelial cells in the HF, which receives signals from epidermal and dermal compartments [23]. Although diverse intercellular signaling pathways including those mediated by Eda/Edar, sonic hedgehog, Notch, TGF-β, BMP, etc. are intricately involved in hair development process, the Wnt/β-catenin signaling pathway plays a central role in the hair morphogenesis and cycling during both the embryonic stage and adult life [24,25]. In canonical Wnt/β-catenin signaling, the secreted Wnt proteins bind with the Frizzled receptor and a co-receptor of the low-density lipoprotein-related protein (LRP), thereby inactivating glycogen synthase kinase-3β (GSK-3β), which is an enzyme responsible for phosphorylation and ubiquitination-mediated degradation of β-catenin. Wnt-mediated inactivation of GSK-3β, thus, stabilizes β-catenin in cytoplasm. Alternatively, the activation of receptor tyrosine kinase can inactivate GSK-3β via phosphorylation of upstream kinase Akt or extracellular signal regulated kinase (ERK), thereby allowing cytosolic β-catenin to escape ubiquitin-dependent degradation [26]. The stabilized β-catenin binds with T-cell factor (TCF)/lymphoid enhancer factor (LEF). The subsequent nuclear translocation of β-catenin-TCF/LEF causes transcriptional activation of genes involved in the regulation of cell proliferation [27,28]. A transmembrane cargo protein, Wntless (*WI*), causes the secretion of Wnt, which is expressed in developing hair placodes, embryonic epidermis and HF [29]. Figure 2 represents the role of various Wnt proteins in hair growth and regeneration. Among the Wnt family proteins, Wnts 3, 4, and 6 are known as primary Wnts, which are not required for hair placode initiation. On the other hand, Wnts 2, 7b, 10a, and 10b are called secondary Wnts, which participate in HF development [30,31]. Kishimoto et al. generated Wnt3a-overexpressed keratinocyte feeder cells and co-cultured these cells with freshly isolated murine DP cells. The presence of Wnt3a was shown to activate β-catenin and increased hair growth in nude mice receiving skin reconstitution composed of DP and keratinocytes [32].

The concept of the involvement of Wnt signals in follicular development stemmed from the early findings of the presence of nuclear β-catenin in chick feather prior to placode formation [33] as well as the expression of Lef1 in mouse dermis during vibrisia follicle formation [34]. Subsequent studies have reported that genetic ablation of β-catenin gene in epidermis leads to the failure of placode morphogenesis in mice [24], suggesting the importance of Wnt signals in hair development. Andl and colleagues [35] generated transgenic mice having epidermal basal cell-specific ectopic expression of Dickkopf 1 (DKK1), which inhibits Wnt action [36,37,38,39,40] by binding with LRP co-receptor, to evaluate the role of Wnt in HF. This study reported that skin-specific overexpression of DKK1 leads to an early and complete block in the development of skin appendages including all types of hair follicles, thus suggesting the essential roles of WNT proteins in follicle development. A significant increase in DKK1 immunostaining was found in leisonal scalp biopsies taken form patients with androgenic alopecia and alopecia areata as compared to healthy control volunteers [35]. The administration of recombinant human DKK-1 (rhDKK-1) into mouse hypodermis resulted in final hair follicle length and premature onset of catagen, a key feature of male pattern baldness. Conversely, injecting neutralizing DKK-1 antibody delayed the anagen-to-catagen transition in mice. This study also demonstrated that treatment with rhDKK1 negated Wnt-mediated activation of β-catenin, thereby inducing apoptosis in outer root sheath keratinocytes via induction of pro-apoptotic protein Bax [41].

The Wnt protein expression and secretion are regulated by the gene *Wl*, alternatively known as Gpr177. Genetic ablation of epithelial Wls expression inhibited hair placode formation at E14.5, and diminished the expression of placode markers, such as Eda, BMP2, BMP4, and SHH, and that of nuclear β-catenin. Restoration of the stabilized β-catenin expression led to the epithelial placode formation but failed to initiate dermal condensate and dermal papilla formation, suggesting that epithelial Wnt signaling is capable of epithelial hair formation but not inducing a dermal response. These authors further demonstrated that dermis-specific deletion of Wl inhibited β-catenin reporter activity in vitro and reduced dermal cell proliferation, although β-catenin expression in vivo remained unchanged and epithelial placode formation was present [42]. These findings suggest that dermal Wnt may not be vital for epidermal HF activation and indicate the presence of adequate Wnt in epidermis to induce this pathway. In fact, Chen et al. [43] demonstrated that epidermal Wnt can induce β-catenin response in the dermis. Moreover, mice with genetically deleted Wl in the basal layer of epidermis and hair follicle showed a marked hair cycle arrest, which was associated with decreased Wnt/β-catenin signaling in follicular epithelium and dermal papilla as compared with control hair follicles [44]. A previous study also demonstrated that a DP-specific depletion of β-catenin induced an early anagen arrest [45]. In contrast, forced activation of β-catenin signaling caused thickening of the dermis and enlargement of placodes and dermal condensates in mice, resulting in the initiation of epidermal HFs [43].

There are multiple lines of evidence suggesting that various Wnts promote hair cycling and regeneration via the activation of β-catenin signaling. For instance, intradermal injection of Wnt1a-enriched conditioned media from bone marrow mesenchymal stem cells in to depilated mouse skin accelerated HF progression from telogen to anagen, increased the amount of hair, and elevated the expression of hair induction-related genes, such as Lef1, versican, and Gli1 [46]. Wnt10b enhanced the differentiation of cultured primary skin epithelial cells into hair shaft and inner root sheath (IRS) of the HF by stabilizing β-catenin. Moreover, addition of Wnt10b into whisker HF culture in serum-free conditions caused elongation of the hair shaft and incorporation of BrdU in dermal papillary matrix cells via β-catenin stabilization [47]. Li et al. [22] further demonstrated that Wnt10b promotes HF growth by enhancing telogen to anagen switch by activating β-catenin signaling. According to their study, the culturing of mouse vibrissae in medium containing adenoviral-Wnt10b grew faster as compared to that cultured in control medium or medium containing Wnt signaling antagonists. A recent study reported that treatment with macrophage-extracellular vesicle (MAC-EV), which contains high level of Wnt3a and -7b, significantly increased the proliferation of DP cells, which was associated with enhanced expression of β-catenin and the activation of transcription factors Axin2 and Lef1. Moreover, intradermal injection of Wnt3a- and Wnt7b-enriched MAC-EV in male balb/C mice enhanced HF growth in vivo. Moreover, treatment with MAC-EV increased the size of hair shaft in human HF in a short time period [48]. Conversely, Wnt5a, a non-canonical Wnt expressed in bulge and secondary hair germ cells at telogen stage, antagonizes the function of canonical Wnts. Adenovirus-mediated overexpression of Wnt5a in mouse dorsal skin elongated the telogen stage and attenuated anagen entry, which was associated with decreased β-catenin expression [49]. The Wnt5a-mediated inhibition of dermal cell proliferation was reversed by the addition of Wnt3a [50].

Factors that regulate Wnt-β-catenin signaling in HF include androgen [51], BMP [52], soluble frizzled receptor protein-1 (sFRP1), and growth factors [53,54,55]. For example, treatment with dihydrotestosterone attenuated Wnt3a-induced TCF/Lef reporter activity in DP cells derived from androgenic alopecia [51]. Conditional inactivation of the BMP type IA receptor in epithelial stem cells located in the bulge region of HF induced overproduction of HF stem/progenitor cells and the genetic manipulation of the BMP signaling pathway caused the activation of β-catenin, suggesting that BMP activity regulates the HF cycle by antagonizing Wnt/β-catenin pathway [15]. The adenovirus-mediated overexpression of BMP6 attenuated the proliferation of HFSC and retarded telogen–anagen transition of HF in C57 mice in vivo. While overexpression of BMP6 inhibited the expression of Wnt10b in HFSC, adenoviral Wnt10b treatment in telogenic HF reduced the number of BMP6-positive cells as compared to control. Since Wnt10b acts as an activator and BMP6 functions as an inhibitor of β-catenin signaling, a cellular balance between these opposing factors are required to regulate the telogen to anagen transition of hair follicles [52]. EGF treatment increased the nuclear translocation of β-catenin, and induced the expression of Wnt10b, β-catenin, EGF receptor, SOX9, and that of a number of follicle-regulatory genes, such as survivin, Msx2, and SGK3. Blocking β-catenin activation by XAV-939 abrogated EGF-induced proliferation of outer root sheath cells [56]. Beyond Wnt stimulation, the β-catenin signal transduction is regulated by other factors, such as growth factor receptor signaling. Topical treatment with FGF enhanced hair growth by earlier onset of anagen and prolongation of mature anagen phase in telogenic C57BL/6 mice. Moreover, FGF treatment caused an early induction of β-catenin and Shh in mouse HF [21]. To assess the role of FGF and Wnt antagonist sFRP1 in outer root sheath cells and DP cells, Zhang et al. isolated these cells from mink skin and cultured them in the presence of FGF10, sFRP1, or both. Whereas FGF10 increased nuclear localization of β-catenin and stimulated the proliferation of both outer root sheath cells and DP cells, sFRP1 treatment attenuated β-catenin activation and dampened the proliferation of these cells. Co-treatment with both FGF10 and sFRP1 showed moderate effect on cell proliferation, migration, and β-catenin level, suggesting that FGF10 and sFRP1 regulate hair cycle by activating or inhibiting β-catenin signaling, respectively [57]. Connective tissue growth factor (CCN2), a matricellular protein, functions as a physiologically relevant suppressor of HF formation, and maintains stem cells quiescence. The expression of CCN2 is restricted to DP and outer root sheath cells. While deletion of CCN2 in DP cells and outer root sheath cells shortened telogen phase and increased the number of HFs, loss of CCN2 in vivo resulted in increase in K15-positive epidermal stem cells with high β-catenin protein level and β-catenin-dependent reporter gene activity. Conversely, overexpression of CCN2 in keratinocytes destabilized β-catenin signaling and reduced cell proliferation [55].

A recently conducted gene ontology analysis of microarray gene expression data collected from bald frontal and haired occipital scalps of five men with androgenic alopecia revealed that all downregulated genes belong to the Wnt and TGF-β signaling pathways, while upregulated genes fall under the oxidative stress pathway [58]. Exposure to high dose ionizing radiation, a genotoxic insult that leads to hair loss, induces HF dystrophy, which is associated with a p53-dependent suppression of Wnt signaling. Augmenting Wnt signaling abrogated the suppressive effect of p53 and increased the ectopic progenitor cell proliferation, thereby preventing IR-mediated hair loss [59].

## 4. Progress in Developing Therapy for Hair Loss by Targeting Wnt/β-Catenin Signaling

The well-defined role of Wnt/β-catenin activation in hair morphogenesis has led researchers to find activators of this signaling pathway as potential therapy for hair loss treatment. Until now, many natural as well as synthetic compounds have been reported to promote hair regrowth via activation of Wnt/β-catenin signaling (Table 1). The following section is a brief account of recent updates in searching novel drug candidates that activates Wnt/β-catenin as a mechanism of hair growth promotion. Tocotrienol, a vitamin E analog, is a well-known antioxidant. Ahmed et al. examined the hair growth promoting effect of a tocotrienol-rich formulation (TRF). Topical application of TRF (5 mg/cm^2^) on depilated dorsal skin of healthy or diabetic mice markedly induced epidermal hair follicle development and early anagen induction, as evidenced by change of skin color from pink to black. Mechanistically, TRF inhibited the expression of epidermal E-cadherin, resulting in the increased nuclear translocation of β-catenin and its interaction with Tcf3. Co-treatment of mouse skin with a pharmacological inhibitor of β-catenin arrested TRF-induced anagen hair cycling [60]. The seed (nut) oil of *Prunus mira* Koehne, a Tibetan medicinal plant, has long been used traditionally to care hairs and eyebrows. High performance liquid chromatography-based chemical analysis of *P. mira* nut oil revealed the presence of a-tocopherol, β-sitosterol, vitamin E, linoleic acid, and oleic acid. Topical application of *P. mira* nut oil (0.47–60.26 mg) on depilated telogenic (pink color) dorsal skin of C57BL/6 mice significantly increased the dermal thickness, number of HFs, hair weight, and hair length as compared to that of minoxidil. *P. mira* nut oil also increased the mRNA and protein expression of β-catenin. Skin irritation assay showed that application of *P. mira* nut oil on New Zealand rabbit skin caused mild erythema, which disappeared within 1 h of treatment [61]. Truong et al. demonstrated that red ginseng oil and its constituents linoleic acid and β-sitosterol promoted hair re-growth in testosterone-treated mouse skin through early induction of anagen phase after once daily application for 28 days. These products elevated the expression of β-catenin, Lef-1, Sonic hedgehog, Smoothened, Gli-1, Cyclin D1, and Cyclin E in the testosterone-treated mice [62].

Several terpenoid compounds have been reported to exhibit hair growth promotion by activating Wnt/β-catenin signaling pathway. Loliolide, a monoterpenoid hydroxyl lactone obtained from brown or red algae, increased the viability of human hair follicle dermal papilla (HDP) cells without causing any cytotoxicity until a dose of 100 μg/mL. Loliolide (20 μg/mL) significantly increased the size of HDP spheroids in 3D culture and enhanced the expression of growth factors, such as vascular endothelial growth factor (VEGF), insulin-like growth factor (IGF), and keratinocyte growth factor (KGF) in HDP spheroids. These effects of loliolide was mediated through increased nuclear localization of β-catenin and enhanced activation of TCF/Lef transcriptional activity via activation of Akt and phosphorylation-dependent inactivation of GSK-3β. Pharmacological inhibition of Akt abrogated loliolide-induced mRNA expression of Wnt5a and Lef1, suggesting that the β-catenin activation by loliolide was mediated through Akt activation. Moreover, loliolide upregulated the expression of DP signature genes, such as alkaline phosphatase (ALP), versican, and BMP2, in HDP spheroids. These studies further demonstrated that loliolide-treated HDP spheroids secretes VEGF, IGF, and KGF, and the culturing of HaCaT keratinocytes using conditioned media from loliolide-treated HDP spheroids increased the proliferation and migration of keratinocytes 9 [65]. Likewise, treatment of sinapic acid, a cinnamic acid derivative, induced proliferation of human DPC cells and increased the expressions of the several growth factors, such as IGF-1 and VEGF, which are required for hair growth. Sinapic acid also increased level of phospho-GSK-3β and β-catenin via activation of Akt in DPCs [66]. Costunolide, a sesquiterpene lactone present in various medicinal plants, holds diverse biological activities including antioxidant, anti-inflammatory, and anti-neoplastic activities [67]. A recent study from our laboratory demonstrated that custonolide (1 or 3 µM) increased the viability of human hair follicle dermal papilla (HDP) cells and reduced testosterone-induced 5-a-reductase activity. Treatment with costunolide also increased the expression of β-catenin and its targeted cell proliferative gene cyclin D1, and increased phosphorylation Rb in HDP cells. Moreover, application of costunolide onto the depilated back of C57BL/6 mouse for 15 days caused significant hair growth in mice. Analysis of post-autopsy dermal layer revealed marked higher diameter and depth of HF in costunolide -treated mouse skin [68].

According to current literature, several naturally occurring flavonoids and chalcones also stimulate hair growth by targeting Wnt/β-catenin pathway. Baicalin is a glycosyloxyflavone abundantly present in many medicinal plant species. Xing et al. [69] examined the hair growth promoting effect of baicalin in Balb/c-nu mice grafted with dermal and epidermal cells isolated from C57BL/6 mouse. According to this study, the mouse skin was treated two weeks post grafting with baicalin (50 or 100 µmol), a positive control drug minoxidil (100 µmol), and IWR1 (100 µmol), which is an inhibitor of β-catenin, once daily for 28 days. As compared to minoxidil treatment, baicalin induced marked hair growth in Balb/c-nu mice, whereas IWR1 caused negligible hair formation. Analysis of skin tissues revealed that baicalin, compared to minoxidil, induced the mRNA and protein expression of Wnt3a, 5a, frizzled 7, desheveled-2, β-catenin, Lef1, and DPC signature gene ALP, while reduced that of GSK-3β. Although treatment with IWR-1 diminished expression of Wnta3a, 5a, β-catenin, and ALP, as well as increased GSK-3β, it would be more rational to see if combination of treatment with IWR-1 and Baicalin could abrogate the induction of growth promoting signals [69]. Silibinin, a flavonoid isolated from the medicinal plant *Silybum marianum*, is an antioxidant and anti-inflammatory compound known to cure various skin disorders. Silibinin induced human hair follicle dermal papilla (HDP) cells spheroid formation in 3D culture. Treatment with silibinin elevated the phosphorylation of Akt, increased the expression of Wnt5a and Lef1, and promoted the TCF/Lef reporter activity. Co-treatment with an Akt inhibitor abrogated silibinin-induced Akt phosphorylation and TCF/Lef activity. In addition, silibinin increased the mRNA levels of DPC markers ALP, FGF7, and BMP2, suggesting the potential of silibinin in promoting hair growth [70].

A well-known herbal medicinal resource is *Ginkgo biloba* extract, which is widely used for the treatment of various cardiac and neuronal diseases [78]. Among the major bioactive constituents of *Ginko boliba*, ginkgolide B and bilobalide have been shown to increase the viability of mink dermal papilla cells, which was associated with the phosphorylation of Akt, ERK (for only ginkolide), and GSK-3β. These ginkolides, at a concentration of 50 µM, also elevated the expression of β-catenin and downregulated the expression of DKK1 in DP cells. However, the hair growth promoting potential of these compounds in vivo is yet to be reported [71]. Morroniside, an iridoid glycoside extracted from *Cornus officinalis*, possesses diverse biological activities. Treatment of outer root sheath cells isolated from human scalp with morroniside at 1 or 10 µM concentrations elevated nuclear localization of β-catenin and increased the proliferation and migration of these cells in culture, which was diminished upon co-treatment with DKK1. Morroniside also upregulated the expression of Wnt10b, β-catenin, and lef1 in outer root sheath cells. Injection of morroniside (100 µM) in depilated mouse skin increased the skin thickness and immunohistochemical analysis revealed the elevated expression of β-catenin in epidermis, outer root sheath cells, and matrix during telogen–anagen transition. The compound markedly reduced catagen entry as compared to control [72]. Several other plant extracts, such as extracts of *polygonum multiflorum* [73], *Aconitie ciliare* tuber [75], *Thuja orientalis* [76], *Malva verticilata* [77], and rice bran extract [79], have been reported to promote mouse skin hair growth or the proliferation of human DPCs via the activation of β-catenin signaling. Our laboratory has reported that topical application of 3-Deoxysappanchalcone (3-DSC), a bioactive constituent of *Caesalpinia sappan* L. (Leguminosae), increased the expression of cyclin-dependent kinase-4 (Cdk4), FGF, and vascular VEGF, and promoted anagen phase hair growth in C57BL/6 mice via STST3 activation. This study also demonstrated that 3-DSC reduced the phosphorylation of β-catenin and increased the total β-catenin level without affecting its mRNA script [80]. In our recent human trial of a hair tonic formulated by using extract of *Broussonetia papyrifera* showed promising hair growth potential. Treatment with the extract decreased the phosphorylation of β-catenin and increased expression of total β-catenin in human DPC cells.

## 5. Conclusions

Despite enormous effort in developing a safe and effective therapy for hair loss, there are few options available for clinical practice. The limited efficacy and remarkable side effects profiles of the existing therapies have led to extensive research on understanding hair biology, especially the molecular mechanisms underlying hair loss and the hair follicle regeneration process. Of the diverse intracellular signaling pathways implicated in hair biology, Wnt/β-catenin signaling plays a key role in stimulating hair follicle stem cells and hair regeneration. Thus, efforts have been made to develop therapies for hair loss treatment by targeting this signaling pathway. While this mini review is designed to shed light on the role of Wnt/β-catenin signaling in hair growth promotion and summarizing the current status of drug development by targeting this pathway, there have been other molecular targets (e.g., growth factors, notch, Gli, STAT5, etc.) for designing drugs for hair loss treatment [81,82]. As it is beyond the scope of this article, a detailed discussion on the molecular pathways other than Wnt/β-catenin is not addressed here. Interestingly, until now, many natural products, which are activators of Wnt/β-catenin, have been identified as potential candidates for further development of therapies for hair loss. Large scale human clinical trials to ascertain the safety and efficacy of these potential Wnt/β-catenin activators are warranted, which may lead to the development of a clinically effective therapy for hair loss treatment in the future.

## Figures and Tables

**Table 1:** Characteristics of natural products acting on the Wnt/b-catenin signaling pathway.

Compound/Extract (Structure)	Experimental Model	Experimental Findings	Ref
**Tocotrienol** 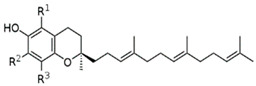	Incubation of HaCaT keratinocytes with 1 μM for 24 h Topical application (5 mg/cm^2^ skin) for 21 days in male C57BL/6 mice Human trial: 35 volunteers, tocotrienol capsule (50 mg) twice daily for 8 months	Reduced expression of E-cadherin. TCF3 activation by increasing nuclear translocation of β-catenin. The db/db mice showed an increase in the number of anagen hair follicles. Inhibited the expression of epidermal E-cadherin, Oct4, Sox9, Klf4, c-Myc, and Nanog. Increased 34% in hair counts. (Baseline 284.8 ± 111.3 vs. after treatment 383.1 ±120.9)	[60,63]
**3,4,5-tri-O-caffeoylquinic acid** 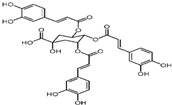	Treatment of human DPCs with 10 μM for 48 h Topical application (1% aqueous solution) in C3H mice	Significantly enhanced the 60% ATP content. Enhanced the hair growth by approximately 40%, 80%, and 120% at Days 14, 20, and 30 after treatment. Activation of Wnt/β-catenin signaling pathway.	[64]
**Loliolide** 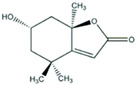	Human DPCs treated with 20 μg/mL for 48 h	Increased spheroid size. Increased growth factor (IGF-1, VEGF.) Hair induction through the activation of AKT-mediated Wnt signaling.	[65]
**Sinapic acid** 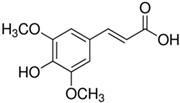	Human hair follicle DPCs incubated with 10, 50, and 100 μM of the compound	Increased hair count to the levels of 118.3%, 119.2%, and 150.5%. Activation of β-catenin, p-GSK-3β and growth factor (IGF-1, VEGF).	[66]
**Costunolide** 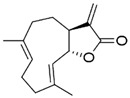	Human hair follicle DPCs treated with 3 μM for 48 h. C57BL/6 mice treated topically with 200 µL of costunolide (3 µmol/L) twice daily for 15 days,	Promoted the cell proliferation through WNT-β-catenin, hedgehog-Gli activity and inhibition of the TGF-β1-Smad pathway. Promoted significant hair growth in mice. The diameter and depth of hair follicles are significantly higher.	[67,68]
**Baicalin** 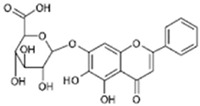	Skin and epidermal cells were isolated from the skin of a C57BL/6 mouse grafted in a 1:1 ratio by incising a wound on the back of a Balb/c-nu mouse and treated with 50 μmol or 100 μmol for 28days.	Significantly increased TCF/LEF reporter activity. Increased ALP activity and stimulates the expression of Wnt3a, Wnt5a, frizzled 7 and disheveled 2. Activation of Wnt/β-catenin signaling and increased of ALP, IGF-1 and VEGF. Significantly increased hair shafts.	[69]
**Silibinin** 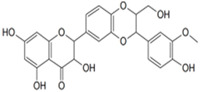	Human hair follicle DPCs treated with 10 μM for 48 h	Increase of 13.53% in the viability of cells, enhances the formation of DP spheroids in vitro, Activates the Wnt/β-catenin signaling pathway and increased the expression of Wnt5a and Lef1. Elevated the phosphorylation of Akt.	[70]
***Ginkgo biloba* extract** 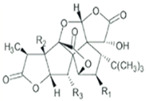	Human hair follicle DPCs treated with 0, 25, 50, and 100 μM for 48 h.	Increased cell growth on time and dose dependent activities of Akt, ERK1/2, and β-catenin.	[71]
**Morroniside** 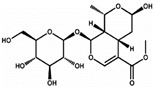	Outer root sheath cell (ORSC) and DP cell incubated 10 µM for 72 h. C57BL/6 mice were injected into mouse HFs on post-depilation day: 100 µM in 100 μL (100 μL over 3 or 6 days)	Significantly increased proliferation by 1.3-fold and increased ORSC migration. Upregulated Wnt10b, β-catenin and lef1 and regulates cell growth and development partly through the Wnt/β-catenin signaling pathway. Accelerated hair cycling from telogen to anagen Delay catagen progression.	[72]
***polygonum multiflorum* extract** 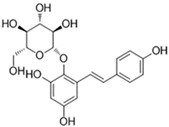	C57BL6/N mics shaved on the dorsal skin were treated with a dose of 4.7 mg per 12 cm^2^ as topically for 1–4 weeks.	Hair length was significantly longer, promotes hair growth by inducing anagen phase in resting hair follicles. Induction of β-catenin and Sonic hedgehog (Shh).	[73,74]
**Prunus mira Koehne** 2,3,5,4-tetrahydroxystilbene-2-*O*-*β*-d-gluoside (TSG) Seed(nut) oil (include α-tocopherol, Vitamin E, β-sitosterol, linoleic acid, and Oleic acid.)	Crude oil 60.26 mg (cm^2^·day)^−1^ and 1/2 diluted in C57BL/6 mice were administered once every 7 days and observed on 21 days.	Increasing Wnt 10 b, β-catenin mRNA and protein expression, and GSK-3 β protein expression. Significant increase in hair length, hair growth rate, and hair weight.	[61]
**Red ginseng oil (RGO)**	Topical treatment with 10% RGO for 28 days on C57BL/6 mice	Restored hair regenerative. Upregulated Wnt/β-catenin and Shh/Gli pathways-mediated expression of genes such as β-catenin, Lef-1, Sonic hedgehog, Smoothened, Gli-1, Cyclin D1, and Cyclin E.	[62]
***Aconitie ciliare* tuber** Natural Plant extracts	Human hair follicle DPCs and rat vibrissa dermal papilla cells (RvDP) treated with 5–50 µg/mL for 24 h. After dorsal skin, C57BL/6 mice were treated with topical treatment at 10 mg/mL for 5 weeks.	Significant cell growth at 10 µg/mL. Activates the Wnt/β-catenin signaling pathway by enhancing β-catenin transcription. Induced early telogen-to-anagen phase conversion of hair follicles.	[75]
**Thuja orientalis** Natural Plant extracts (Platycladus orientalis)	C57BL6/N mice shaved on the dorsal skin were treated with 5.05 mg/cm^2^/day as topically for 21 days	Promotes hair growth, hair length and number of hair follicles. Induction of β-catenin and Shh.	[76]
***Malva verticilata*** Natural Plant extracts (Chinese mallow)	Human hair follicle DPCs treated with 20 µg/mL for 72 h.	Increased cell proliferation. Increased β-catenin and increased transcription levels (IGF-1, KGF, VEGF and HGF).	[77]
